# Oncology education for family medicine residents: a national needs assessment survey

**DOI:** 10.1186/s12909-020-02207-0

**Published:** 2020-08-27

**Authors:** Steven M. Yip, Daniel E. Meyers, Jeff Sisler, Keith Wycliffe-Jones, Edward Kucharski, Christine Elser, Claire Temple-Oberle, Silvana Spadafora, Paris-Ann Ingledew, Meredith Giuliani, Sara Kuruvilla, Nureen Sumar, Vincent C. Tam

**Affiliations:** 1 Department of Oncology, Faculty of Medicine, University of Calgary, Tom Baker Cancer Centre, 1331 29th St NW, Calgary, AB T2N 4N2 Canada; 2 Cancer Care Manitoba, University of Manitoba, 675 McDermot Ave, Winnipeg, MB R3E 0V9 Canada; 3 Department of Family Medicine, University of Calgary, 1403 29 St NW, Calgary, AB T2N 2T9 Canada; 4 Department of Family and Community Medicine, University of Toronto, 500 University Ave, Toronto, ON M5G 1V7 Canada; 5 Radiation Medicine Program, Princess Margaret Cancer Centre, University of Toronto, 610 University Ave, Toronto, ON M5G 2C1 Canada; 6 Northern Ontario School of Medicine, Ramsey Lake Rd, Sudbury, ON P3E 6H6 Canada; 7 Vancouver Cancer Center, B.C. Cancer, University of British Columbia, 600 West 10th Ave, Vancouver, BC V5Z 4E6 Canada; 8 London Regional Cancer Centre, University of Western Ontario, 800 Commissioners Road East, London, ON N6A 5W9 Canada

**Keywords:** Oncology, Cancer, Medical education, Teaching, Residency, Family medicine, General practitioner, Family doctor

## Abstract

**Background:**

This study aimed to determine the current state of oncology education in Canadian family medicine postgraduate medical education programs (FM PGME) and examine opinions regarding optimal oncology education in these programs.

**Methods:**

A survey was designed to evaluate ideal and current oncology teaching, educational topics, objectives, and competencies in FM PGMEs. The survey was sent to Canadian family medicine (FM) residents and program directors (PDs).

**Results:**

In total, 150 residents and 17 PDs affiliated with 16 of 17 Canadian medical schools completed the survey. The majority indicated their programs do not have a mandatory clinical rotation in oncology (79% residents, 88% PDs). Low rates of residents (7%) and PDs (13%) reported FM residents being adequately prepared for their role in caring for cancer patients (*p* = 0.03). Residents and PDs believed the most optimal method of teaching oncology is through clinical exposure (65% residents, 80% PDs). Residents and PDs agreed the most important topics to learn (rated ≥4.7 on 5-point Likert scale) were: performing pap smears, cancer screening/prevention, breaking bad news, and approach to patient with increased cancer risk. According to residents, other important topics such as appropriate cancer patient referrals, managing cancer complications and post-treatment surveillance were only taught at frequencies of 52, 40 and 36%, respectively.

**Conclusions:**

Current FM PGME oncology education is suboptimal, although the degree differs in the opinion of residents and PDs. This study identified topics and methods of education which could be focussed upon to improve FM oncology education.

## Background

Approximately 40% of Canadians will develop cancer in their lifetime and 30% will die from cancer [1]. Due to the high prevalence of cancer, nearly all family physicians (FPs) are involved in the screening, management, post-treatment surveillance, and palliation of cancer patients. According to the 2010 National Physician Survey, approximately 84% of family physicians reported having managed patients with cancer, and these rates are expected to increase in the future with the increasing incidence of cancer [2]. More Canadian FPs have narrowed their scope of practice to focus on cancer care as evidenced by the fact that the Canadian Association of General Practitioners in Oncology (CAGPO) now consists of 146 members [3].

Despite the growing need for cancer care in family practice, there is little focused oncology teaching in undergraduate medical education and postgraduate family medicine (FM) residency training in Canada [4, 5]. In 2009, one survey study of family medicine program directors and academic co-ordinators found that only 12.5% of respondents reported more than 1 week of cancer education in their FM training program and 75% indicated that only 1 to 5% of their entire FM curriculum focused on cancer [4]. In another study, cancer education was also thought to be inadequate in their training program by 57% of FM residents [5]. These residents believed that cancer is the least adequately taught subject compared to all other medical subspecialty-related diseases [5]. None of these training programs had a mandatory oncology rotation or a formal oncology curriculum, but 2 programs did report having oncology objectives for their residents [5].

In the United States, a survey of 77 FM and internal medicine residents from several schools found that only 5% of the participating residents rated themselves as very knowledgeable in long-term follow-up care of adult cancer survivors [6]. Current postgraduate training education opportunities to enhance knowledge in cancer prevention and control are limited [7]. In another Kentucky and Tennessee survey of FM, internal medicine, obstetrics and gynecology (OB/GYN), and preventive/occupational medicine residents, approximately one quarter of respondents (25.6%) did not feel capable of discussing current cancer-related care guidelines [8].

Though the current survey data indicate inadequate education in oncology for FPs in North America, a rigorous analysis of the existing state of oncology education in Canadian FM residency programs has yet to be performed. The purpose of this Canadian national survey study was to assess in detail the current state of oncology education in Canadian FM residency training programs and to determine the optimal topics and potential curricular interventions for educating family medicine residents regarding cancer.

## Methods

This Canadian national needs assessment survey study was approved by the University of Calgary Conjoint Health Research Ethics Board. Data collection was completed from May 1 to August 31, 2017.

### Survey and data collection

The postgraduate medical education (PGME) surveys were designed to evaluate ideal and current oncology teaching, topics, objectives and competencies in FM PGME. Two separate surveys were developed specifically for FM residents and FM program directors (PD) (see Additional file 3 for the survey questions).

The surveys were initially developed by a group of Canadian physicians, including: a FM residency PD, a chair and sitting member of the Family Physician Cancer Care Committee of the College of Family Physicians of Canada and Physicians, a general practitioner oncologist, five medical oncologists, two radiation oncologists, and one surgical oncologist. Prior to distribution, the surveys were assessed for face and content validity by this group and pilot-tested with a group of 5 general practitioners. All surveys were available in both English and French.

A self-administered web-based survey was created to determine the opinions of FM residents and PDs regarding oncology education in their residency training programs. Residents in FM training programs are classified into postgraduate years, including year 1 (PGY-1), year 2 (PGY-2), and sometimes year 3 (PGY-3). The first component assessed demographics and asked whether a formal oncology curriculum is currently taught at the respondents’ FM residency program and whether a set of learning objectives or competencies are provided to the residents. The survey then inquired about currently-taught oncology topics, teaching methods employed, and perceived adequacy of the education in oncology. The next component of the survey included questions surrounding the optimal teaching methods for oncology education oncology to FM residents and the most important oncology topics to be learned (using free text and drop-down menu response options). Finally, respondents were asked about the usefulness of a national set of standardized learning goals, objectives and competencies in oncology for FM residency training programs.

Canadian FM residency PDs from all 17 FM residency programs were contacted by e-mail and asked to complete the survey. Some family medicine training programs were identified to have more than one PD (e.g. one in charge of the urban program and another in charge of the rural program). In such cases, each PD was asked to complete the survey. The PDs were also asked to forward a web link to the resident version of the survey to all of their FM residents. PDs were asked to indicate the total of number of residents who would receive the survey in order to determine response rates. We attempted to enhance the response rate by sending subsequent reminders and offering coffee cards to those who completed the survey. A second reminder invitation was sent to PDs.

### Statistical analysis

The survey was conducted using the website www.surveymonkey.com (© 1999–2019 SurveyMonkey). Following completion, aggregate data was transferred to a password-protected computer for analysis. Statistical analysis was performed using the Microsoft Excel software application (version 15.0: Microsoft Corp., Redmond, WA, U.S.A.). The response frequencies and descriptive statistics were calculated where appropriate. Fisher’s exact test was used to examine the difference between PDs’ and residents’ responses.

## Results

**Fig. 1:**
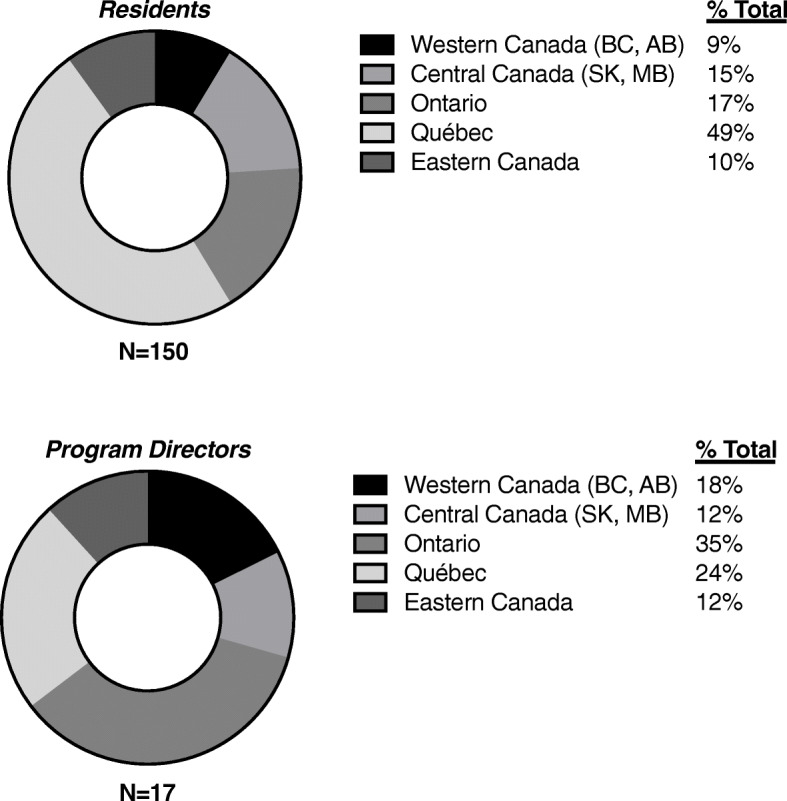
Geographic distribution of survey respondent

The demographic characteristics of the FM resident and PD respondents are shown in Table 1. A total of 19 family medicine PDs were identified from the 17 Canadian medical schools and 17 completed the PD survey (response rate = 87%). They represented 16 of the 17 medical schools with FM training programs. The 17 program directors agreed to distribute the resident survey link to a total of 902 FM residents, of which 150 completed the survey (response rate = 17%). Figure 1 shows the geographic distribution of respondents across Canada. A total of 17 of 19 FM programs were represented with responses from PDs and/or residents from all areas of Canada. Thus, this sample is representative of the general FM PD population. Although it was not possible to disseminate the survey to one Western Canadian FM training program, due to logistic barriers, we were still able to obtain survey responses from this institution’s program directors.

**Table 1:** General characteristics of survey respondents

Characteristic	***Surveyed group [n (%)]***	
	Residents (***N*** = 150)	PDs (***N*** = 17)
**Gender**		
Male	36 (24%)	5 (29%)
Female	111 (74%)	12 (71%)
Other	3 (2%)	0 (0%)
**Program year**		
PGY-1	32 (21%)	N/A
PGY-2	113 (75%)	N/A
PGY-3	5 (3%)	N/A
**Number of years in practice**		
< 10	N/A	2 (12%)
10–20	N/A	4 (24%)
> 20	N/A	11 (65%)
**Area of current clinical practice**^a^		
Comprehensive care	116 (77%)	12 (71%)
Focused in oncology	3 (2%)	1 (6%)
Focused in other area	31 (21%)	4 (24%)
**Location of current practice/training**		
Urban	103 (69%)	5 (29%)
Rural	25 (17%)	0 (0%)
Both	22 (15%)	12 (71%)

^a^Anticipated area of practice listed for residents

Table 2 summarizes key findings with regards to the current state of oncology teaching in Canadian FM training programs (all raw response data from residents and program directors are available in Additional file 1 and Additional file 2, respectively). Based on these survey results, it appears that none of the FM training programs currently have a mandatory oncology rotation. Five PDs (29%) report having oncology learning objectives and competencies, but many residents are unaware that these exist (*p* = 0.04). Very few PDs (13%) and residents (7%) reported that residents are being adequately prepared for their role in caring for cancer patients (*p* = 0.03). We did not observe any significant trends regarding specific responses by university site.

**Table 2:** Status of current oncology education in family medicine training programs

Question	***Surveyed group [n (%)]***		
	Residents (***N*** = 150)	PDs (***N*** = 17)	Fisher’s exact test ***P*** value
**Is there a mandatory oncology clinical rotation/block?**			0.90
Yes	10 (7%)	0 (0%)	
No	118 (79%)	15 (88%)	
Unsure	2 (1%)	0 (0%)	
No Response	20 (13%)	2 (12%)	
**Are there oncology learning objectives/competencies?**			0.04
Yes	17 (11%)	5 (29%)	
No	66 (44%)	9 (53%)	
Unsure	47 (31%)	1 (6%)	
No Response	20 (13%)	2 (12%)	
**Is the oncology education provided adequate?**			0.27
Yes	10 (7%)	3 (18%)	
No	120 (80%)	12 (71%)	
Unsure	0 (0%)	0 (0%)	
No Response	20 (13%)	2 (12%)	
**Does your PGME adequately prepare you to care for cancer patients?**			0.03
Yes	11 (7%)	2 (13%)	
No	74 (49%)	3 (18%)	
Unsure	45 (30%)	10 (59%)	
No Response	20 (13%)	2 (12%)	

The most common instructional method was informal clinical teaching around cases during a rotation by family physicians, as reported by 88% of PDs (Table 3). However, only 49% of residents reported receiving formal oncology teaching (e.g. lecture-based, problem-based learning) in their family medicine clinics. Oncology teaching through didactic lectures and small group/case-based learning were reported by 76% of PDs. Yet, only 36 to 37% of residents reported learning oncology through these teaching methods. PDs and residents agreed that the optimal methods for teaching oncology to FM residents is preferentially through clinical exposure, followed by didactic teaching, and also small group/case-based learning (Table 4).

**Table 3:** Current methods of oncology teaching to family medicine residents

Method of oncology teaching	***Surveyed group [n (%)]***		
	Residents (***N*** = 150)	PDs (***N*** = 17)	Fisher’s exact test ***P*** value
**Didactic lectures from family physicians?**			0.006
Yes	56 (37%)	13 (76%)	
No	62 (41%)	1 (6%)	
Unsure	11 (7%)	1 (6%)	
No Response	21 (14%)	2 (12%)	
**Didactic lectures from oncologists?**			0.16
Yes	23 (15%)	4 (24%)	
No	102 (68%)	9 (53%)	
Unsure	4 (3%)	2 (12%)	
No Response	21 (14%)	2 (12%)	
**In clinic by family physicians?**			0.007
Yes	73 (49%)	15 (88%)	
No	47 (31%)	0 (0%)	
Unsure	7 (5%)	0 (0%)	
No Response	23 (15%)	2 (12%)	
**Clinical rotation with general practitioner in oncology (GPO)?**			0.0001
Yes	25 (17%)	10 (59%)	
No	99 (66%)	3 (18%)	
Unsure	6 (4%)	2 (12%)	
No Response	20 (13%)	2 (12%)	
**Clinical rotation with oncologist (medical, surgical, radiation)?**			< 0.0001
Yes	20 (13%)	13 (76%)	
No	104 (69%)	2 (12%)	
Unsure	5 (3%)	0 (0%)	
No Response	21 (14%)	2 (12%)	
**Small group/case-based learning?**			0.01
Yes	54 (36%)	13 (76%)	
No	63 (42%)	2 (12%)	
Unsure	12 (8%)	0 (0%)	
No Response	21 (14%)	2 (12%)	
**Online/web-based learning?**			0.35
Yes	8 (5%)	2 (12%)	
No	105 (70%)	13 (76%)	
Unsure	16 (11%)	0 (0%)	
No Response	21 (14%)	2 (12%)	
**Independent learning?**			0.96
Yes	33 (22%)	3 (18%)	
No	73 (49%)	8 (47%)	
Unsure	23 (15%)	3 (18%)	
No Response	21 (14%)	2 (12%)

**Table 4:** Optimal method of teaching oncology to family medicine residents

Optimal Method of Teaching	***Surveyed group [n(%)]***		
	Residents (***N*** = 150)	PDs (***N*** = 17)	Fisher’s exact test ***P*** value
Clinical Exposure	84 (65%)	12 (80%)	1.000
Didactic Teaching/Lectures from Specialists	37 (29%)	5 (33%)	
Small Group/Case-Based Learning	32 (25%)	4 (27%)	

*N.B. Survey respondents were allowed to list up to three responses, so responses do not add up to 100%

Table 5 shows that based upon the survey results, the most important oncology topics for FM residents to learn in descending order of mean perceived importance, accompanied by the perceived prevalence of current teaching of each topic. The topics thought to be most important by residents with a mean rating of 4.5 out of a 5-point Likert scale or higher were: performing pap smears, cancer screening, breaking bad news, cancer prevention, and approach to a patient with increased risk of cancer and palliative care. The PDs generally agreed that these topics are most important, but also included providing psychosocial support and performing a skin biopsy as areas of importance. There was general consensus between PDs and residents that all of these topics are being taught to residents (ranging between 87 and 100% frequency, per item). However, the following important topics were taught with relatively lower frequency rates, according to PDs: appropriate referral to cancer specialists (73%), post-treatment surveillance for recurrence (47%), managing common treatment side effects (47%), managing common cancer complications (40%), and management of long term complications from treatment (13%).

**Table 5:** Oncology topic perceived importance and prevalence of current teaching

Topic	***Surveyed Group***			
	Residents (***N*** = 150)		PDs (***N*** = 17)	
	***Mean Importance***^a^	***Currently taught***	***Mean Importance***^a^	***Currently taught***
Performing pap smears	4.9	99%	5.0	100%
Screening for common cancers	4.9	100%	4.9	100%
Breaking bad news	4.8	96%	5.0	93%
Cancer prevention	4.7	95%	5.0	93%
Approach to patient with increased risk of cancer	4.7	92%	4.7	93%
Palliative care	4.6	89%	5.0	100%
Approach to diagnosis	4.5	89%	4.7	93%
Providing psychosocial support	4.4	75%	4.8	87%
Performing skin biopsy	4.3	85%	4.9	100%
Appropriate referrals to cancer specialists	4.2	52%	4.3	73%
Post-treatment surveillance for recurrence	4.1	36%	4.0	47%
Managing common complications	4.0	40%	3.6	40%
Managing common treatment side effects	4.0	39%	4.4	47%
Epidemiology of common cancers	3.9	80%	3.2	67%
Prognosis of common cancers	3.8	44%	3.6	20%
Management of long term complications from treatment	3.7	18%	3.4	13%
Management of common cancers	3.5	36%	3.6	40%
Approach to cancer treatment	3.1	34%	3.7	64%
Approach to staging cancer	2.9	24%	2.5	20%
Performing fine needle biopsy	2.8	15%	2.9	21%
Performing bone marrow biopsy	1.8	3%	1.5	0%

^a^*Likert scale out of 5, 5 = very important, 1 = not important*

According to PDs, the five cancer disease sites viewed to be of greatest educational importance for FM residents are breast (100%), lung (93%), colorectal (80%), prostate (73%), and cutaneous (73%). Residents stated that breast (93%), lung (90%), colorectal (83%), prostate (73%), and cutaneous (30%) cancers were of greatest interest and educational value to them.

When asked whether a set of standardized national oncology learning goals, objectives and competencies for family medicine would be useful 62% of residents and 53% of PDs agreed. Only 3 and 12%, respectively, disagreed while the others were unsure.

## Discussion

This study is the first to describe in detail the current state of FM residency training oncology education in Canada. There is general agreement among the residents and PDs who responded that current oncology education in family medicine does not adequately prepare the residents for their role in caring for cancer patients as family physicians. PDs believe that oncology education is delivered in FM clinics, didactic lectures and small groups at a much higher rate compared to the residents. There is better agreement between PDs and residents regarding the optimal methods to teach oncology to FM residents and the most important oncology topics to be taught.

The main result of our study is consistent with previous studies, which have shown that oncology education in non-oncology medical training programs is thought to be suboptimal by the majority of FM residents and PDs [4, 5]. In contrast to Tam et al.’s sample of 7 PDs and 93 residents, our study uniquely differs from the previous publication [5], in that there was participation from 89% of PDs and a larger number of family medicine residents (*n* = 150), who represent nearly all of Canada’s family medicine training programs. It is interesting to note that previously in 2011, 43% of FM PDs and 14% of residents believed oncology education was inadequate, which is much higher than the 18 and 7%, respectively, found in this current survey. Although it is difficult to draw a comparison between these two studies, this may indicate that only modest progress has been achieved to improve cancer education for these FM residents over the last 6 years despite the findings from the previous study.

Findings from a study in the United States appear to reflect a similar trend regarding FM and internal medicine residency education in oncology, where 81% of residents expected to care for cancer survivors in their future practice, but only 27% of the residents reported formal education in adult cancer survivorship care [6]. This resulted in only 13% feeling comfortable in their role as a primary care provider for adult cancer survivors. These findings are congruent with our Canadian FM residency training results, which indicate that the deficiency in FM oncology education is not unique to Canada and may be potentially reflective of the state of FM oncology education in other countries.

The present study also details the importance of specific oncology topics to be included in FM education and suggests the perceived optimal methods of teaching these topics in the FM residency training. It appears that additional topics, such as appropriate referrals to cancer specialists, post-treatment surveillance of cancer, managing common cancer complications and common treatment side effects, are topics of perceived educational importance that are infrequently taught. FM training programs can likely improve oncology education for their residents by focussing on increased teaching of these specific topics.

This study indicates a need to improve FM education surrounding cancer care, and it has identified areas that should be focused on, both in terms of topics and teaching format. In particular, there is an absence of an oncology module in FM education. FM educators should consider educational reform to ensure that comprehensive oncology education, which addresses the current needs of patients and the healthcare team, is provided consistently to all FM residents across Canada. This can be achieved by implementing a set of standard oncology competencies for graduating FM residents could help residency programs address the gap in training identified in this study. The previous study also found that there was broad support for a standard set of oncology objectives among FM PDs and FM residents (71 and 93%, respectively) [5]. This current study shows continued support for the development of oncology education competencies, which would help inform the FM training programs and the FM residents of the essential oncology topics and experiences to be learned during residency training. It is our understanding that there is currently no formal oncology training for the majority of family medicine residents in Canada. Most programs do not have specific learning objectives for oncology. However, the College of Family Physicians of Canada (CFPC) considers cancer to be one of its priority topics, for the purposes of assessment, and the CFPC has described an accompanying set of “Key Features” that relate to this topic which focus on cancer prevention, screening, follow-up and support, assessment of patient’s ability to cope, inquiry about side effects or complications, monitoring of recurrence, and prognosis discussion [9]. Expanding upon CanMEDS-FM as an existing curriculum framework for Family Medicine in Canada, as well as the CFPC priority topic of cancer (along with its key features), Canadian national oncology education competencies, goals, and objectives for family medicine residents could certainly be created using a similar Delphi process that was used to develop the Canadian Oncology Goals and Objectives for Medical Students in 2014 through a national Delphi process [9–13]. A national curriculum that can be directly implemented into FM residency programs is beyond the scope of this study. Further research engaging all stakeholders – family physicians, FM residents, PDs, and general practitioner oncologists – is necessary to develop a comprehensive national curriculum, in order for this work to translate into true educational reform. Nevertheless, it is hoped that this study sets the groundwork necessary for future efforts in this setting. To help facilitate future efforts for educators in this field, our current findings and what is already described by the CFPC suggest that course contents and learning objectives may aim to focus on the following areas: performing pap smears, cancer screening, breaking bad news, cancer prevention, approach to a patient with increased risk of cancer and palliative care, providing psychosocial support, performing a skin biopsy, appropriate referrals to cancer specialists, post-treatment surveillance for recurrence, managing common treatment side effects, managing common cancer complications, and management of long term complications from treatment, follow-up and support, assessment of patient’s ability to cope, and prognosis. Since all family medicine training programs are not identical and most residents learn oncology from their clinical preceptors, it is most practical to have goals and objectives where the residents can look through and find any gaps in their oncology knowledge and pursue the appropriate learning opportunities.

### Limitations

Given the ambitious national focus of this survey study, the results from the FM resident survey may be limited by self-selection bias, where FM residents, who have more of an interest in cancer care, were more likely to respond. Also, it was not possible to disseminate the survey to one Western Canadian FM training program, due to logistic barriers. We were still able to summarize the opinions and experiences of 150 FM residents, which is the largest cohort in the published literature on this topic, despite the lower response rate. Although the PD respondents are representative of the population of PDs, the sample of residents were identified to be more predominantly female (74%), compared to Canadian Residency Match (CaRMS) data, which shows approximately 60% of those residents matched to FM programs are female from 2017 to 2019 [14–16]. Hence, this difference in gender distribution may not be as representative of the FM residency population. The lower response rate by FM residents may also be mitigated by the fact that we can be confident in the accuracy of our results from the PD survey. For this group, there was a high response rate of 87%, and the responses represented 88% of the FM residency training programs across Canada. An additional limitation is the fact that there are differences in responses by FM residents from the same training program, which may be secondary to recall bias or having different experiences with various clinical preceptors during their training.

## Conclusions

Currently, Canadian family medicine residency oncology education is suboptimal, although the degree to which this occurs differs in the opinion of residents and program directors. This study identifies specific topics and methods of education as well as highlighting areas that could be focused upon in any curriculum design to improve FM oncology education. This study sets the groundwork upon which we may further engage stakeholders to develop and determine standardized oncology learning goals and competencies for family medicine residents that can be implemented in their training programs.

## Supplementary information

**Additional file 1.** Residents Survey Raw Data. Survey responses from family medicine residents.

**Additional file 2.** Program Director Survey Raw Data. Survey responses from Program Directors.

**Additional file 3.**

## Data Availability

Additional files 1 and 2 provide the raw data of survey responses.

## Abbreviations

FM PGME: Family medicine postgraduate medical education
FM): Family medicine
PDs: Program directors
PGME: Post graduate medical education
PGY-1), year 2 (PGY-2), year 3 (PGY-3: Postgraduate year 1
