# Supplementary material for: Oncology education for family medicine residents: a national needs assessment survey
Source: BMC Med Educ. 2020 Aug 27;20:283. doi: 10.1186/s12909-020-02207-0 (PMC7457241; doi:10.1186/s12909-020-02207-0)
Supplement: Supplementary file 3 — Additional file 3. [file 12909_2020_2207_MOESM3_ESM.docx]

**Appendix A: Family Medicine Resident Survey**

- 1. Are you a resident in a Canadian family medicine residency training program?


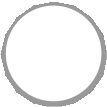
 Yes
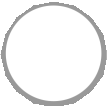
 No

- 2. What is your gender


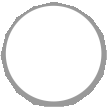
 Female


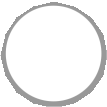
 Male


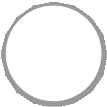
 Transgender


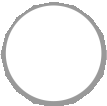
 Prefer not to say


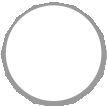
 Other (please specify)

- 3. What is your current level of training


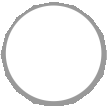

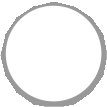
 PGY
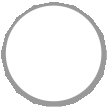
 PGY-2 PGY-3

- 4. With which Canadian medical school are you currently affiliated?


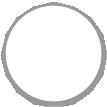
 University of British Columbia
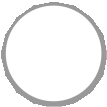
 University of Calgary


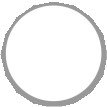
 University of Alberta


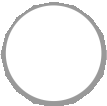
 University of Saskatchewan
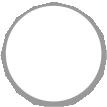
 University of Manitoba


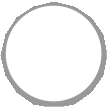
 Northern Ontario School of Medicine


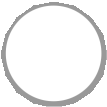
 University of Western Ontario


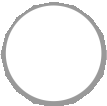
 McMaster University
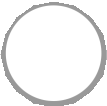
 University of Toronto
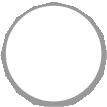
 Queen's University
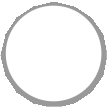
 University of Ottawa
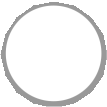
 McGill University


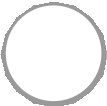
 Universite de Montreal
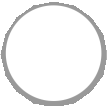
 Universite Laval


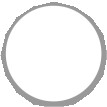
 Universite Sherbrooke
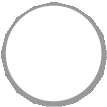
 Dalhousie University
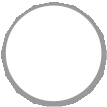
 Memorial University

- 5. Is your family medicine training program best described as urban or rural? An urban area is defined as having a population of at least 1,000 and a density of 400 or more people per square kilometre. All territory outside an urban area is defined as rural area.


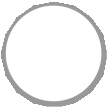
 Urban
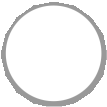
 Rural
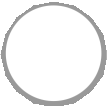
 Both

- 6. Do you plan to practice in an urban or rural setting in the future?


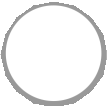

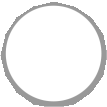
 Urban
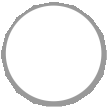
 Rural
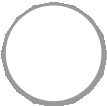
 Both

Unsure

- 7. Which of the following best describes your future planned clinical practice?

Family physician (Comprehensive care)

Family physician (With a focussed practice of any part, but not oncology) Family physician (With a focussed practice in oncology (e.g. GPO/FPO))

- 8. Is there a mandatory oncology clinical rotation/block, or a scheduled mandatory horizontal/longitudinal clinical educational experience in cancer care for family medicine residents in your program?


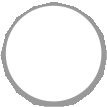
 Yes
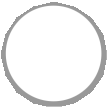
 No


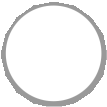
 Unsure

If yes, how many weeks (please specify)

- 9. Is there a standardized set of oncology LEARNING OBJECTIVES or competencies for family medicine residents in your program?


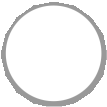

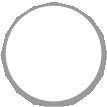
 Yes
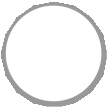
 No

Unsure

- 10. Please consider the setting in which cancer topics are taught during your family residency training program.

Are cancer topics taught in this setting?


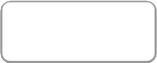


Didactic lectures from family physicians, including general practitioner oncologists/family physician oncologists (GPO/FPO)

Didactic lectures from oncologists


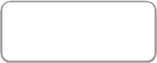


In clinic by family physicians


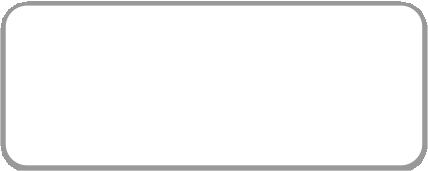
Clinical rotation

with general practitioner

oncologists/family
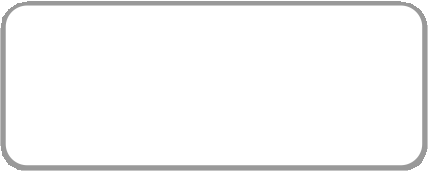
 physician oncologists

(GPO/FPO)


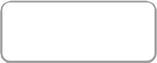


Clinical rotation with oncologists (surgical/radiation/medical oncologists)

Small group or Problem-

Based or Case-Based
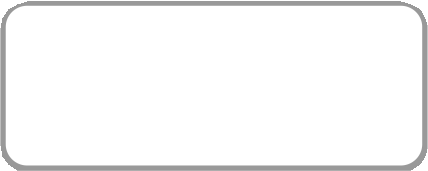
 Learning


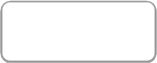


Online/web-based teaching

Independent learning with

a list of resources and
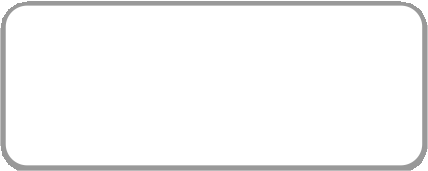
 learning objectives

Other setting (please specify)

- 11. What is the optimal way to teach oncology to family medicine residents? Please refer to the above options in question 10.
- 12. Do you believe the amount of oncology education provided to residents in your family medicine residency training program is adequate?

1 Too little 2 3 Ideal 4 5 Too much


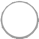

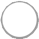

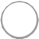

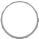

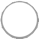


-

- 13. Do you believe that the oncology education provided in your family medicine residency training program has adequately prepared you to care for cancer patients as a family physician?

1 No, I do not feel it prepared me to manage cancer

patients 2


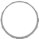

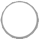

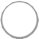

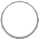

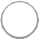


-

3 Not sure, I think it prepared me to manage cancer

patients 4

5 Yes, I definitely feel well prepared to manage cancer patients

Additional comments (please specify)

1. DIAGNOSIS & TREATMENT

Please consider whether the following topics were taught during the course of your family medicine training program and how important it is that these topics are taught in family medicine training programs.

Was the topic taught during the course of your family medicine training program?


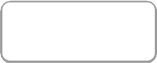

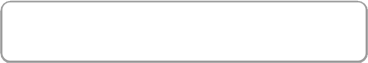


Cancer prevention

How important it is that these topics are taught in family medicine training programs?

Assessment and

approach to patients at


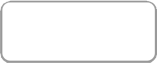

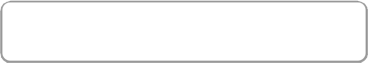


Epidemiology of common cancers (e.g. prostate, lung, breast, colon, skin)

increased risk of cancer

Screening for cancer (e.g. cervical, breast, colon)


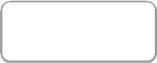

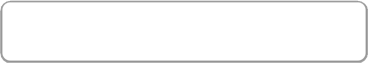


Approach to diagnosis in patient with suspected cancer (e.g. approach to breast lump or solitary pulmonary nodule)

General

approach to staging cancers


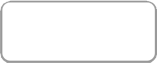

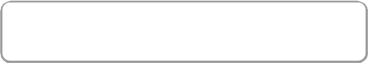


General approach to cancer treatment

Appropriate referrals to specialists in oncology (medical oncology, radiation oncology, surgical oncology, palliative care)


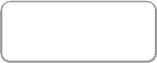

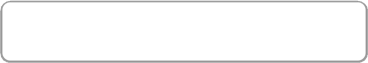


Palliative care for cancer patients

Management of common

cancers (e.g. prostate,
 lung, breast, colon, skin)

1. PROCEDURES

Please consider whether the following topics were taught during the course of your family medicine training program and how important it is that these topics are taught in family medicine training programs.

How to perform skin punch biopsies


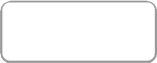

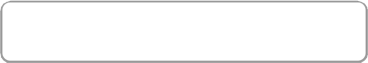


How to perform fine needle biopsies

Was the topic taught during the course of your family medicine training program?


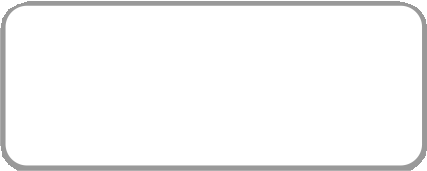


How important it is that these topics are taught in family medicine training programs?


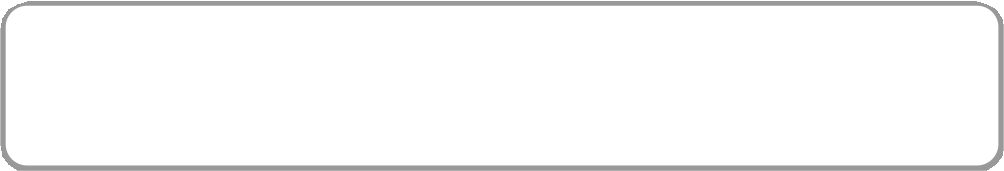


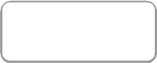

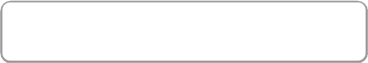


How to perform bone marrow biopsies


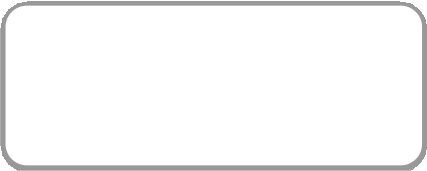

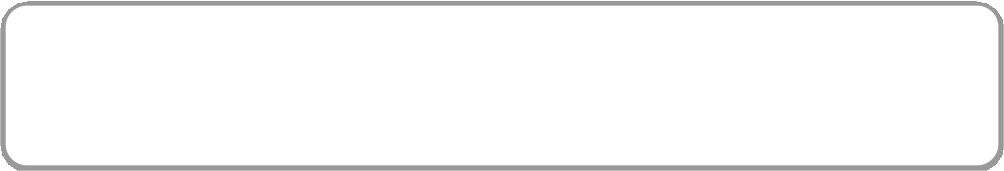
How to perform pap smears

1. COMPLICATIONS, PROGNOSIS and COMMUNICATION

Please consider whether the following topics were taught during the course of your family medicine training program and how important it is that these topics are taught in family medicine training programs.

How to manage common cancer treatment side effects/adverse events


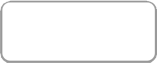

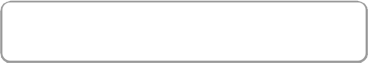


How to manage common complications of cancer

Was the topic taught during the course of your family medicine training program?


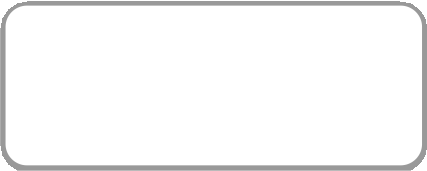


How important it is that these topics are taught in family medicine training programs?


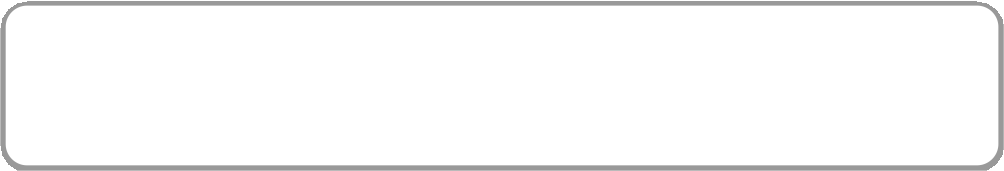


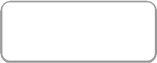

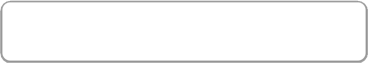

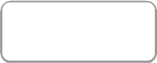

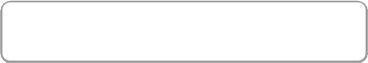

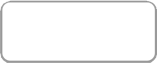

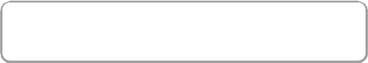


Prognosis of common cancers

How to break bad news

Management of long- term complications/toxicities from cancer treatment

Post-treatment

surveillance/screening for cancer recurrence


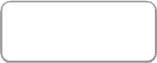

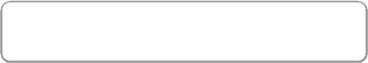


How to provide cancer patients with psychosocial support

- 17. If cancer-specific teaching was offered, which disease site(s) would you be interested in learning about (e.g. lung cancer, colon cancer, breast cancer, etc.) (List up to 5)

18. Are there any other oncology topics not listed above that should be taught during the course of a family medicine training program?

- 19. Please indicate how useful you think a national standardized set of oncology learning goals, objectives and competencies for family medicine resident training would be.

1 Not useful 2 3 Somewhat useful 4 5 Very useful


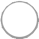

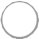

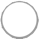

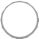

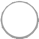


-

**Appendix B: Family Medicine Program Director Survey**

- 1. Are you a program director for your family medicine residency training program?

Yes No

- 2. What is your gender


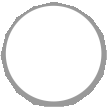
 Female


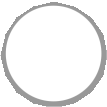
 Male


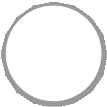
 Transgender


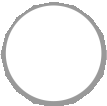
 Prefer not to say


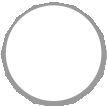
 Other (please specify)

-
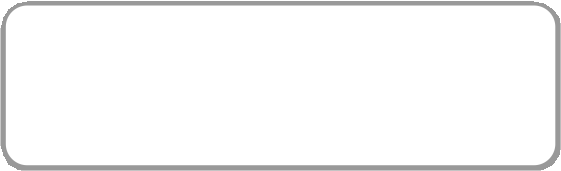
3. How many years ago did you graduate from your own family medicine residency training program?

* 4. Which best describes your involvement in academic family medicine?


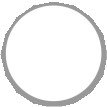
 Academic teacher

Academic researcher

Academic teacher and researcher

- 1. Which best describes your current clinical practice?

Family physician (Comprehensive care)

Family physician (With a focused practice of any part, but not oncology)

Family physician (With a focused practice in oncology (e.g. GPO/FPO))

- 6. With which Canadian medical school are you currently affiliated?

University of British Columbia University of Calgary

University of Alberta

University of Saskatchewan University of Manitoba

Northern Ontario School of Medicine

University of Western Ontario

McMaster University University of Toronto Queen's University University of Ottawa McGill University

Universite de Montreal Universite Laval

Universite Sherbrooke Dalhousie University

Memorial University

* 7. Is your family medicine training program best described as urban or rural? An urban area is defined as having a population of at least 1,000 and a density of 400 or more people per square kilometre. All territory outside an urban area is defined as rural area.

Urban Rural Both

- 8. Do you believe that the oncology education you received during your family medicine residency training adequately prepared you to care for cancer patients as a family physician?
  - 1. No, I do not feel it prepared me to manage cancer

patients 2

-

3. Not sure, I think it prepared me to manage cancer

patients 4

5. Yes, it definitely prepared me well to

manage cancer patients

Additional comments (please specify)

- 9. Is there a mandatory oncology clinical rotation/block, or a scheduled mandatory horizontal/longitudinal clinical educational experience in cancer care for family medicine residents in your program?

Yes No

Unsure

If yes, how many weeks (please specify)

* 10. Is there a standardized set of oncology LEARNING OBJECTIVES or competencies for family medicine residents in your program?

Yes No

Unsure

- 11. Please consider the setting in which cancer topics are taught during your family residency training program.

Are cancer topics taught in this setting?

Didactic lectures from family physicians including general practitioner oncologists/family physician oncologists (GPO/FPO)

Didactic lectures from oncologists

In clinic by family physicians

Clinical electives

with general practitioner

oncologists/family physician oncologists

(GPO/FPO)

Clinical electives with oncologists (surgical/radiation/medical oncologists)

Small group or Problem-

Based or Case-Based Learning

Online/web-based teaching

Independent learning with

a list of resources and learning objectives

Other setting (please specify)

- 12. What is the optimal way to teach oncology to family medicine residents? Please refer to the above options in question 11.

* 13. Do you believe the amount of oncology education provided in to residents in your family medicine residency training program is adequate?

1 Too little 2 3 Ideal 4 5 Too much

-

- 14. Do you believe that the oncology education provided in your family medicine residency training program has adequately prepared trainees to manage cancer patients as family physicians?

1 No, our graduating residents are not prepared to manage cancer

patients 2

3 Not sure, I think our residents can adequately manage

cancer patients 4

5 Yes, our graduating residents are definitely well prepared to manage cancer patients

-

15. DIAGNOSIS & TREATMENT

Please consider whether the following topics are taught during the course of your family medicine training program and how important it is that these topics are taught in family medicine training programs.

Is the topic taught during the course of your family medicine training program?

How important is that these topics are taught in family medicine training programs?

Cancer prevention

Assessment and

approach to patients at

Epidemiology of common cancers (e.g. prostate, lung, breast, colon, skin)

increased risk of cancer

Screening for cancer (e.g. cervical, breast, colon)

Approach to diagnosis in patient with suspected cancer (e.g. approach to breast lump or solitary pulmonary nodule)

General

approach to staging cancers

General approach to cancer treatment

Appropriate referral to specialists in oncology (medical oncology, radiation oncology, surgical oncology, palliative care)

Palliative care of cancer patients

Management of common

cancers (e.g. prostate, lung, breast, colon, skin)

16. PROCEDURES

Please consider whether the following topics are taught during the course of your family medicine training program and how important it is that these topics are taught in family medicine training programs.

How to perform skin punch biopsies

How to perform fine needle biopsies

Is the topic taught during the course of your family medicine training program?

How important it is that these topics are taught in family medicine training programs?

How to perform bone marrow biopsies

How to perform pap smears

1. COMPLICATIONS, PROGNOSIS and COMMUNICATION

Please consider whether the following topics are taught during the course of your family medicine training program and how important it is that these topics are taught in family medicine training programs.

Is the topic taught during the course of your family medicine training program?

How important is that these topics are taught in family medicine training programs?

How to manage common cancer treatment side effects/adverse events

How to manage common complications of cancer

Prognosis of common cancers

How to break bad news

Management of long- term complications/toxicities from cancer treatment

Post-treatment

surveillance/screening for

How to provide cancer patients with psychosocial support

cancer recurrence

Other (please specify)

- 18. If cancer specific teaching was offered, which disease site(s) do you think your residents would be interested in learning about (e.g. lung cancer, colon cancer, breast cancer, etc.) (List up to 5)

19. Are there any other oncology topics not listed above that should be taught during the course of a family medicine training program?

20. Please indicate how useful you think a national standardized set of oncology learning goals, objectives and competencies for family medicine resident training would be.

1 Not useful 2 3 Somewhat useful 4 5 Very useful

-
